# Essential role of multimodality imaging in the diagnosis of a rare high-grade primary cardiac intimal sarcoma

**DOI:** 10.1093/ehjcr/ytag223

**Published:** 2026-03-20

**Authors:** Mohamud A Mohamud, Axucillia Moyo, Kunal Kothari, Rui He, Qiong Zhao

**Affiliations:** Inova Schar Heart and Vascular Institute, Inova Fairfax Hospital, 3300 Gallows Rd, Falls Church, VA 22042, USA; Inova Schar Heart and Vascular Institute, Inova Fairfax Hospital, 3300 Gallows Rd, Falls Church, VA 22042, USA; Inova Schar Heart and Vascular Institute, Inova Fairfax Hospital, 3300 Gallows Rd, Falls Church, VA 22042, USA; Inova Schar Heart and Vascular Institute, Inova Fairfax Hospital, 3300 Gallows Rd, Falls Church, VA 22042, USA; Inova Schar Heart and Vascular Institute, Inova Fairfax Hospital, 3300 Gallows Rd, Falls Church, VA 22042, USA

**Keywords:** Echocardiography, Intimal sarcoma, Primary cardiac sarcoma, Myxoid

## Abstract

**Background:**

Primary cardiac intimal sarcoma is an exceptionally rare and aggressive malignant tumour that often mimics benign cardiac lesions, particularly atrial myxoma. Accurate diagnosis requires multimodality imaging and histopathological confirmation.

**Case summary:**

A 58-year-old woman presented with palpitations, exertional dyspnoea, and recurrent syncope. Echocardiography and CT revealed a large, polypoid left atrial mass attached to the interatrial septum, initially suggestive of atrial myxoma. Due to haemodynamic compromise, urgent surgical resection was performed. Histopathology demonstrated a high-grade intimal sarcoma with myxoid stroma and MDM2 gene amplification. Initial staging showed no metastases; however, tumour recurrence in the left atrium occurred within 2 months. Cardiac MRI confirmed recurrent disease with delayed enhancement. Despite chemotherapy and subsequent radiotherapy for brain metastases, the tumour progressed, and the patient died 7 months after diagnosis.

**Discussion:**

This case highlights the diagnostic challenge of distinguishing benign from malignant cardiac tumours when echocardiography and computed tomography are used in isolation. Although echocardiography is the first-line modality for detecting cardiac masses, it provides limited tissue characterization, and CT offers limited soft-tissue contrast with non-specific enhancement patterns. In contrast, cardiac magnetic resonance imaging provides superior soft-tissue resolution and multiparametric tissue characterization, allowing identification of heterogeneous signal intensity, necrosis, and infiltrative margins that favour malignancy. In this case, CMR findings supported the diagnosis of intimal sarcoma rather than myxoma. Given the aggressive biology and poor prognosis of intimal sarcoma despite multimodal therapy, early multidisciplinary evaluation in a specialized cardio-oncology centre is essential.

Highlights

- Intimal sarcoma is an extremely rare undifferentiated pleomorphic sarcoma that usually arises in the pulmonary artery, less frequently in the aorta or its branches.
- Cardiac intimal sarcoma may be divided into myxoid and non-myxoid types, and myxoid histology of intimal sarcoma may be associated with MDM2 gene amplification, displaying a polypoid appearance mimicking myxoma, Echocardiography plays an essential role of initial evaluation and multimodality imaging helps to differentiate between the two tumours.

Learning points

1. To understand the unique characteristics of the clinical course, Echo and Cardiac CT/MRI imaging and histological findings of primary cardiac intimal sarcomas.
2. To understand the importance of multimodality imaging in making reliable diagnosis of cardiac tumours.

## Introduction

Sarcomas of the cardiovascular system are extremely rare, arising in the aorta, vena cava, or heart. Subtypes include intimal sarcoma/undifferentiated pleomorphic sarcoma (UPS), angiosarcoma, leiomyosarcoma, and undifferentiated sarcoma.^1^ Intimal sarcoma is exceptionally uncommon, usually originating in the pulmonary artery and less often in the aorta or heart. It is highly aggressive, with mean survival of 5–18 months.^2^ Cardiac UPS may resemble intimal sarcoma histologically and molecularly, demonstrating tumour growth within the intima and extension into the vascular lumen.

The WHO Classification of Tumours of the Heart (4th edition) uses ‘intimal sarcoma’ as an alternative designation for some cardiac UPS cases.^3,4^ Fewer than 15 cases of cardiac intimal sarcoma have been reported to date. Presenting symptoms are often non-specific, mimicking other cardiac or systemic conditions, and diagnosis relies on multimodality imaging such as echocardiography (Echo), CT, and cardiac MRI.^5^ Given its aggressiveness, treatment requires a multidisciplinary approach, usually involving surgery, chemotherapy, and radiation, although prognosis remains poor.^6^

We present a case of a 58-year-old woman with a left atrial intimal sarcoma that mimics a myxoma in morphology, illustrating diagnostic challenges and the importance of comprehensive imaging.

## Summary figure

**Figure:**
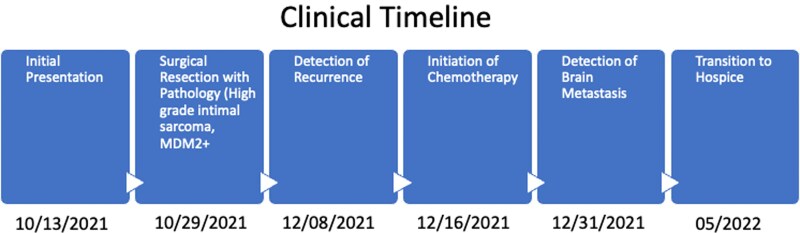


## Case presentation

**Figure 1:**
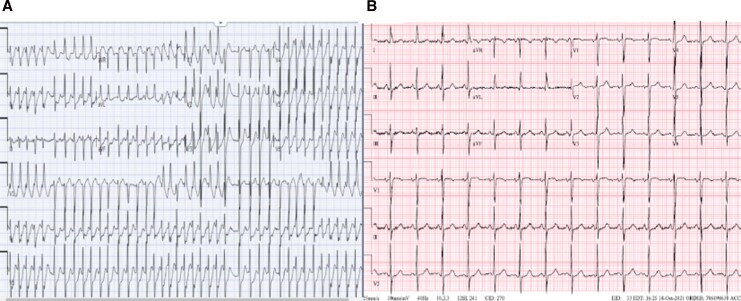
ECG findings. *(A*) Atrial fibrillation. *(B*) Normal sinus rhythm.

A 58-year-old woman with hypothyroidism presented with 2 months of palpitations, progressive exertional dyspnea, and recurrent syncope. On admission, vital signs were within normal limits. Two hours later, she developed atrial fibrillation with a rapid ventricular rate of 234 bpm, which reverted to sinus rhythm after intravenous Diltiazem (*Figure 1A/B*). Examination revealed an irregular rhythm with an early diastolic ‘plop,’ trace pedal edema, but no murmurs. Family and social history were non-contributory.

**Figure 2:**
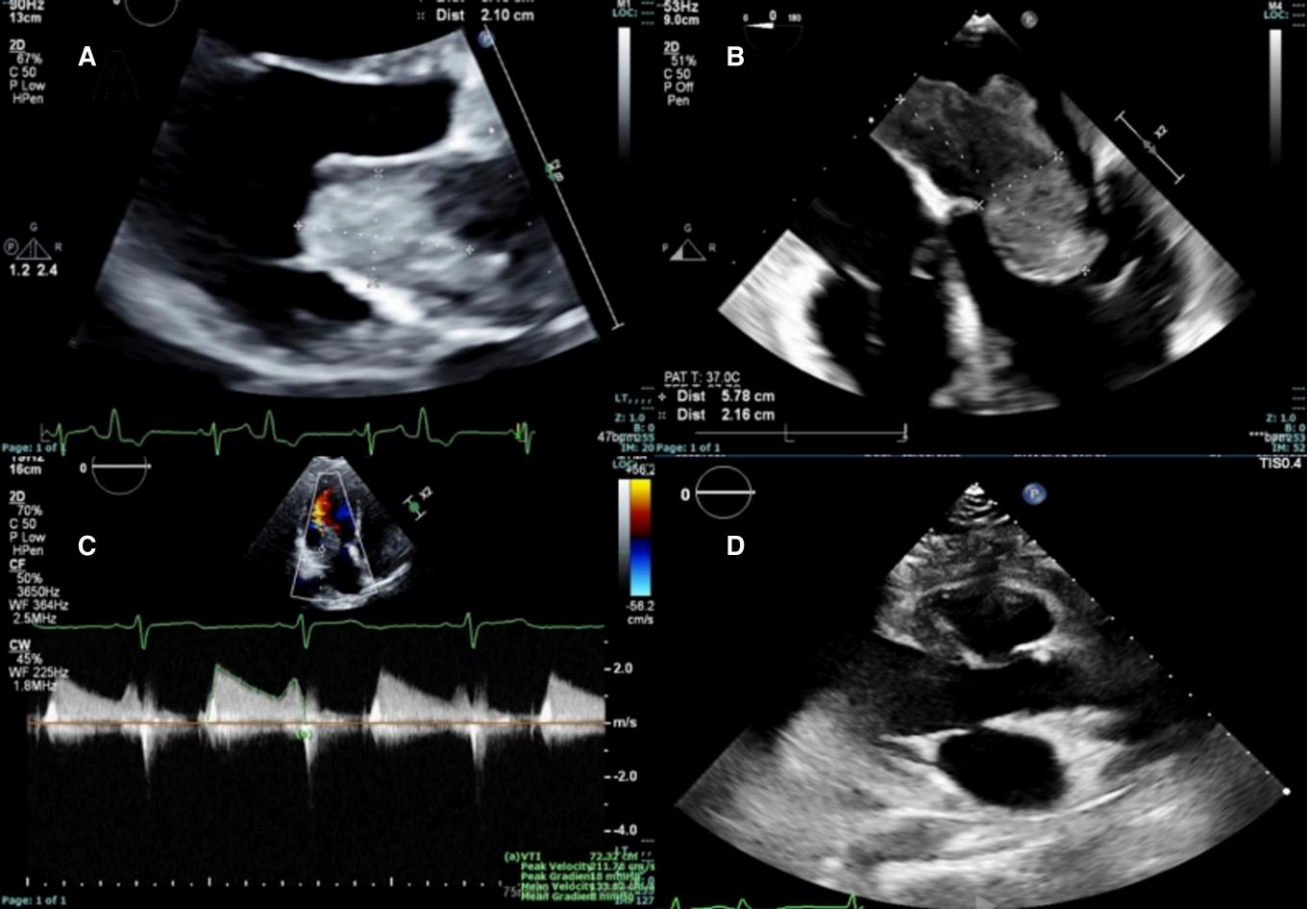
Echocardiogram findings of left atrial mass. *(A*) Parasternal long axis view of transthoracic echocardiogram showing a large polypoid mass in the left atrium obstructing left ventricular inflow during diastole. *(B*) Transoesophageal view of left atrial mass measuring 5.8 cm × 2.2 cm. *(C*) Mean pressure gradient across the mitral valve of 8 mmHg. *(D*) Post-operative echocardiogram in the parasternal long axis view demonstrating complete excision of the left atrial mass.

Initial point-of-care ultrasound showed an echogenic mass in the left atrium. CT angiography, performed to rule out pulmonary embolism, revealed a 5.8 × 3.4 cm heterogeneous left atrial mass attached to the interatrial septum—suspicious for atrial myxoma due to its polypoid appearance. Transthoracic and transoesophageal echocardiography confirmed a large, echogenic mass (*Figure 2A–B*, clips 1–3), encroaching on the anterior mitral leaflet and causing moderate mitral stenosis (mean pressure gradient 8 mmHg) (*Figure 2C*).

Despite features suggestive of myxoma, the tumour’s large size and broad septal attachment raised concern for malignancy.

**Figure 3:**
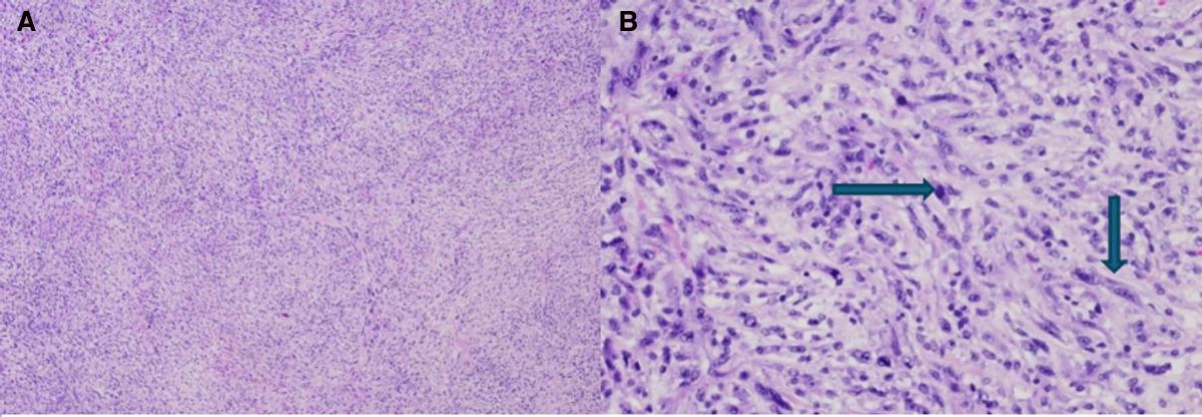
Pathologic features of intimal sarcoma. *(A*) Low magnification view (H&E stain, 4X objective) shows that the tumour is composed of solid sheets of atypical spindle cells and thin-walled blood vessels. *(B*) High magnification view (H&E stain, 20X objective) shows that some atypical spindle cells have hyperchromatic nuclei (vertical arrow). Frequent mitotic features are present (horizontal arrow). Myxoid changes are observed in stroma.

Due to haemodynamic compromise, the patient underwent surgical resection 16 days after initial presentation with a concurrent MAZE procedure. Postoperative Echo confirmed complete excision (*Figure 2D*). Histopathology revealed a high-grade malignant spindle cell sarcoma with myxofibrosarcoma-like features. Cells were pleomorphic with hyperchromatic nuclei embedded in a myxoid stroma containing thin branching vessels (*Figure 3*). Brisk mitotic activity and tumour necrosis were present. Immunohistochemistry was positive for MDM2 (Murine Double Minute 2), and RNA *in situ* hybridization confirmed MDM2 amplification.

The tumour was classified as FNCLCC Grade 3 intimal sarcoma. Genomic testing showed low tumour mutational burden, microsatellite stability, and amplifications of CDK4, KIT, MDM2, and PDGFRA.

**Figure 4:**
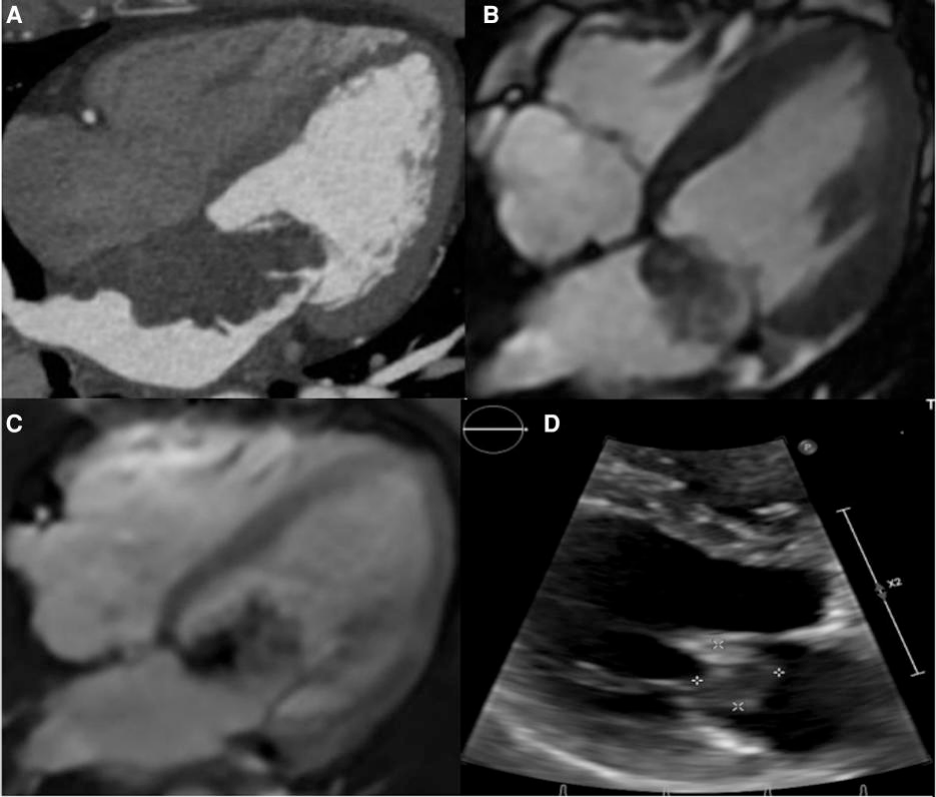
Tumour recurrence on cardiac CT, MRI, and echocardiogram. *(A*) CT image demonstrates a large polypoid left atrial mass along the interatrial septum obstructing the mitral inflow. *(B*) HLA FIESTA image demonstrates a polypoid left atrial mass obstructing the mitral inflow. *(C*) Phase-sensitive HLA LGE image demonstrates the presence of LGE in a polypoid left atrial mass consistent with malignancy. This mass is a biopsy-proven sarcoma. *(D*) Follow-up echocardiogram 2 months after revealed a 1.7 cm × 1.5 cm mass on the anterior leaflet of the mitral valve.

Initial staging showed no metastasis. However, within 40 days after surgery, follow-up Echo revealed a recurrent mass on the mitral valve (1.7 × 1.5 cm) (*Figure 4D*). Cardiac CT and MRI confirmed tumour recurrence involving the interatrial septum and left atrium (*Figure 4A–C*). MRI revealed delayed enhancement—typical of sarcoma.

The patient began chemotherapy with Adriamycin 8 days after recurrence, but the tumour progressed after 1 month. She subsequently developed brain metastases 15 days after initiation of chemotherapy, 79 days from initial presentation, and underwent stereotactic radiosurgery. Adriamycin was replaced with Temozolomide after three cycles, but the disease continued to advance. After evaluation with cardiac surgery, resection of the recurrent mass was not feasible. She died 7 months after her initial diagnosis.

## Discussion

Primary cardiac malignancies are rare (0.0017–0.019% incidence), and among them, primary cardiac sarcomas (PCS)—especially intimal subtypes—are extremely uncommon, with fewer than 15 reported cases.^1,5,7^ Prognosis is poor due to aggressive behaviour and late presentation, with average survival of 9–11 months.^5^

Symptoms are often vague and reflect local tumour effects.^8^ Yin *et al*. identified surgical resection and chemotherapy as independent protective factors, while age correlated with worse outcomes.^6^

Echocardiography remains a key diagnostic tool but has limitations in differentiating benign from malignant lesions. In this case, Echo and CT suggested a benign myxoma. However, the tumour’s broad-based attachment and rapid recurrence post-resection supported a malignant process.

**Figure 5:**
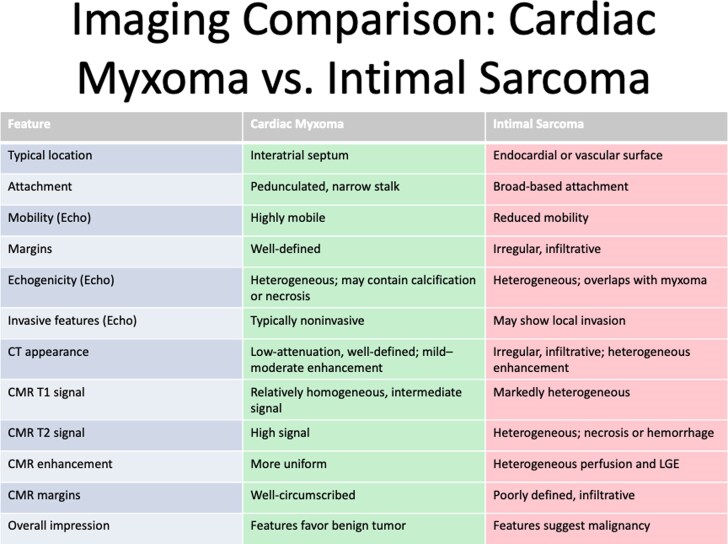
Imaging comparison of Echo, CT, and MRI of myxoma vs. sarcoma.

Intimal sarcoma may be divided into myxoid and non-myxoid types, and myxoid histology of intimal sarcoma may be associated with MDM2 gene amplification.^1^ In this case, the intimal sarcoma was associated with the MDM2 gene application with a myxoid type, showing a polypoid appearance like myxoma, a common benign primary cardiac tumour. Diagnosis based only on Echo and CT findings may lead to delay of the diagnosis and treatment. Echo contrast is commonly used to identify the vascularity of cardiac tumours, typically a characteristic feature of malignant tumours. However, they usually comprise of dense spindle cells, often resulting in low Echo contrast uptake, a common feature of benign tumours. Therefore, it is crucial to use multimodality imaging, including Cardiac CT/MRI to further identify the features of benign vs. malignant tumours, as specified in *Figure 5*. In this case, cardiac MRI showed the characteristic of delayed enhancement in the recurrent tumour.

All of the intimal sarcoma cases with *MDM2* gene amplification demonstrate myxoid stroma, as shown in our case, while a majority of *MDM2* gene amplification-negative intimal sarcoma cases demonstrate non-myxoid sarcomatous histology resembling undifferentiated pleomorphic sarcoma.^1^ On CT, myxomas present as hypodense lesions with a very wean contrast enhancement and calcifications in about 10–20%. On cardiac MRI, myxomas have a heterogeneous appearance with intermediate signal intensity on T1-weighted and higher signal intensity on T2 -weighted FSE sequences.^2^

Primary cardiac sarcomas tend to metastasize to the brain, thus tumour staging, including a brain MRI is important to rule out metastasis.^9^ Due to the rarity of this disease, there is limited evidence on specific therapies.^8^ Current treatment involves surgical resection and neoadjuvant chemotherapy. However, over time, there has not been any significant improvement in mortality in patients with PCS. In their study looking at treatment strategies for PCS, Chan and colleagues highlighted that these patients are complex and require a multidisciplinary cardiac tumour team for a chance of reasonable outcomes.^10^ In another cohort study, Wei-Wu Chen and colleagues assessed PCS outcomes across six multi-national cancer centres. They found no precise optimal management of patients with PCS due to a limited evidence base. In the same study, they found that patients developed recurrent disease despite surgical resection and that survival was poor.^8^ Nowadays, genetic testing has been used more frequently in cancer diagnosis, and genetic targeted treatment such as the potential therapeutic value of targeting MDM2 gene amplification in the myxoid type of intimal sarcoma may improve clinical outcome selected patients in the future.

We present a rare case of primary cardiac intimal sarcoma, myxoid type, associated with MDM2 gene amplification and a polypoid appearance that mimics a left atrial myxoma by Echo and CT. Final diagnosis was established postoperatively through histopathology and supported by cardiac MRI findings of recurrence. This case underscores the diagnostic importance of multimodality imaging in cardiac tumours and the need for clinical suspicion in atypical presentations. Given the aggressive nature and diagnostic complexity of primary cardiac sarcomas, prompt referral to dedicated cardio-oncology centres should be strongly considered, as multidisciplinary expertise and access to advanced imaging, surgical care, and molecularly targeted therapies are crucial for appropriate management and improved prognosis.

## Lead author biography


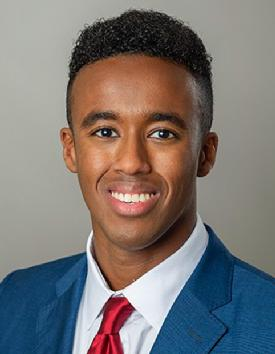


Mohamud Mohamud is an Internal Medicine resident at INOVA Fairfax Hospital in Falls Church, VA. He completed his medical school training at Eastern Virginia Medical School in 2023. He plans to apply to cardiology fellowship training in 2026.

## Supplementary Material

ytag223_Supplementary_Data

## Data Availability

The data underlying this article are available in the article and in its online Supplementary material.

## Notes

**Consent:** The authors confirm that written informed consent for submission and publication of this case report, including images and associated text, has been obtained from the patient’s next of kin in line with COPE guidelines.
